# The antimicrobial activity of free and immobilized poly (diallyldimethylammonium) chloride in nanoparticles of poly (methylmethacrylate)

**DOI:** 10.1186/s12951-015-0123-3

**Published:** 2015-09-24

**Authors:** Luccas Missfeldt Sanches, Denise Freitas Siqueira Petri, Letícia Dias de Melo Carrasco, Ana Maria Carmona-Ribeiro

**Affiliations:** Biocolloids Lab, Departamento de Bioquímica, Instituto de Química, Universidade de São Paulo, Caixa Postal 26077, CEP 05513-970 São Paulo, SP Brazil; Instituto de Química, Universidade de São Paulo, Caixa Postal 26077, CEP 05513-970 São Paulo, SP Brazil; Departamento de Análises Clínicas e Toxicológicas, Faculdade de Ciências Farmacêuticas, Universidade de São Paulo, CEP 05508-900 São Paulo, Brazil

**Keywords:** Biocompatible polymer, Antimicrobial polymer, Antimicrobial nanoparticles, Poly (methylmethacrylate), Particle characterization by light scattering and scanning electron microscopy

## Abstract

**Background:**

Several cationic polymers exhibit a useful antimicrobial property, however the structure–activity relationship still requires a more complete investigation. The main objective of this work is the comparison between the antimicrobial activity and toxicity of free and immobilized poly (diallyldimethylammonium) chloride (PDDA) in biocompatible poly (methylmethacrylate) (PMMA) nanoparticles (NPs).

**Results:**

NPs synthesis by emulsion polymerization is performed over a range of [PDDA] at two methylmethacrylate (MMA) concentrations. The PMMA/PDDA dispersions are characterized by dynamic light-scattering for sizing, polydispersity and zeta-potential analysis, scanning electron microscopy (SEM), plating plus colony forming unities (CFU) counting for determination of the minimal microbicidal concentrations (MMC) against *Escherichia coli*, *Staphylococcus aureus* and *Candida albicans* and hemolysis evaluation against mammalian erythrocytes. There is a high colloidal stability for the cationic PMMA/PDDA NPs over a range of [PDDA]. NPs diverse antimicrobial activity against the microorganisms reduces cell viability by eight-logs (*E. coli*), seven-logs (*S. aureus*) or two-logs (*C. albicans*). The NPs completely kill *E. coli* over a range of [PDDA] that are innocuous to the erythrocytes. Free PDDA antimicrobial activity is higher than the one observed for PDDA in the NPs. There is no PDDA induced-hemolysis at the MMC in contrast to the hemolytic effect of immobilized PDDA in the NPs. Hemolysis is higher than 15 % for immobilized PDDA at the MMC for *S. aureus* and *C. albicans*.

**Conclusions:**

The mobility of the cationic antimicrobial polymer PDDA determines its access to the inner layers of the cell wall and the cell membrane, the major sites of PDDA antimicrobial action. PDDA freedom does matter for determining the antimicrobial activity at low PDDA concentrations and absence of hemolysis.

**Electronic supplementary material:**

The online version of this article (doi:10.1186/s12951-015-0123-3) contains supplementary material, which is available to authorized users.

## Background

The rise of multidrug resistant pathogens is one of the top three threats to global public health listed by the World Health Organization [1, 2]. The biomedical applications of synthetic, biodegradable polymers for the development of anti-infective strategies can overcome instability, side effects, toxicity and frequent dose regimens of drugs [2]. A possible approach to obtain antimicrobial nanoparticles (NPs) involves polymers as carriers of the active substance or the polymer itself as the active substance [3]. Biocompatible and antimicrobial polymers are becoming the materials of choice for developing several novel structures of biomedical importance against infectious diseases [3–7]. There are two established routes to produce polymeric systems with antimicrobial properties. The first assembles by physical interactions the active molecule in a polymer matrix, while the second involves covalent binding of the active substance in the polymer carrier [3]. Polycationic structures usually present high activity, due to mass and charge localization that promotes electrostatic interactions between the cationic structure and the negatively charged bacterial cell membrane, resulting in disruption of the cell membrane and/or wall [8, 9]. Being environment friendly also plays in favor of the usage of quaternary ammonium compounds (QACs) [10]. QACs such as dioctadecyldimethylammonium bromide (DODAB) are miscible with some biocompatible polymers such as poly (methylmethacrylate) (PMMA) in spin-coated films, do not leak from the polymeric matrix and kill bacteria upon contact [11]. Cetyltrimethylammonium bromide (CTAB) is more mobile and leaks out from PMMA films killing bacteria in the bulk solution [12]. PMMA NPs containing CTAB or DODAB synthesized by emulsion polymerization display high bactericidal activity [13]. For obtaining the PMMA NPs, emulsion polymerization involves two steps: nucleation and growth of the particles [14–18]. The available sites for polymerization are the monomer droplets of the methylmetacrylate (MMA) in water dispersed as a microemulsion [19]. Slightly hydrophilic monomers such as MMA result in smaller droplets because MMA at the interface of polymerized particles acts as a co-surfactant [20]. The free radicals generated by the initiator can react with MMA in the micelles and inside the MMA monomer droplets in the aqueous phase, yielding oligoradicals. As the chain length of the oligoradicals increases, they can diffuse into the micelle interior and/or the monomer droplets to proceed with the polymerization [14]. Among the cationic antimicrobial polymers, poly (diallyldimethylammonium) chloride (PDDA) also exhibits the quaternary ammonium moiety as pendant groups in its chemical structure and displays outstanding antimicrobial activity [4, 21–23]. In this work, we synthesize PMMA/PDDA NPs by surfactant-free emulsion polymerization of MMA in the presence of PDDA in order to determine the effect of PDDA immobilization on its activity against microorganisms or red blood cells.

## Results

### Preparation and characterization of PMMA/PDDA NPs

Emulsion polymerization of MMA in the presence of PDDA with azobisisobutyronitrile (AIBN) as the radical initiator is done under N_2_ flow in order to prevent the precocious termination of the free radicals required for the MMA addition reaction and the synthesis of the PMMA particles. Table 1 shows the main physical properties of the dispersions as a function of different feed concentrations of the MMA monomer or the cationic antimicrobial polymer amphiphile (PDDA). Formulations A3, A4, A5, B3, B4 and B5 yield cationic dispersions with high colloidal stability at PDDA concentrations of 3, 4 or 5 mg/mL, respectively, at two different MMA concentrations employed during the particle synthesis 0.56 (named A) or 1.32 M MMA (named B). Thus a formulation A3 means that particle synthesis was carried out with 0.56 M MMA and 3 mg/mL PDDA whereas a formulation B4 means that 1.32 M MMA and 4 mg/mL PDDA were used for particle’s synthesis. In general, for syntheses carried out in the presence of PDDA, the zeta-average diameter (Dz) or mean hydrodynamic diameter varied between 230 and 328 nm while the zeta-potential (ζ) values varied between 62 and 69 mV. All dispersions in Table 1 present long-term colloidal stability. Precipitates or flocs do not occur for as long as 2 years. At [PDDA] <2 mg/mL, precipitation of PMMA/PDDA is visible. Thus the dispersions at and above 3 mg/mL of [PDDA] are stable and selected for further determination of antimicrobial activity. The incorporation of PDDA into the PMMA/PDDA NPs is shown from elemental analysis for nitrogen in the lyophilized dispersions (Table 1).

**Table 1:** Physico-chemical properties of PMMA/PDDA dispersions in aqueous medium at 1 mM NaCl

NPs	D_z_ ± SD (nm)	D ± SD (nm)	ζ (mV)	P	M_w_ (g/mol)	SC (mg/mL)	Np (mL^−1^)	% C	% H	% N
A3	257 ± 2	127 ± 12	66 ± 2	0.06 ± 0.01	1,173,000	5.5 ± 0.4	4.33 × 10^12^	53.8 ± 0.6	8.6 ± 0.1	2.4 ± 0.3
A4	328 ± 2	112 ± 17	67 ± 1	0.03 ± 0.01	1,065,000	10.0 ± 0.7	1.15 × 10^13^	55.4 ± 0.5	8.6 ± 0.1	1.5 ± 0.2
A5	286 ± 2	164 ± 32	69 ± 4	0.02 ± 0.01	2,872,000	4.4 ± 0.2	1.62 × 10^12^	51.5 ± 0.0	9.0 ± 0.2	3.7 ± 0.1
B3	232 ± 1	151 ± 27	62 ± 2	0.07 ± 0.01	1,514,000	7.3 ± 0.4	3.46 × 10^12^	52.5 ± 0.0	8.8 ± 0.3	3.0 ± 0.3
B4	230 ± 1	139 ± 22	67 ± 2	0.04 ± 0.01	2,362,000	5.8 ± 0.4	3.47 × 10^12^	50.3 ± 0.0	9.2 ± 0.4	4.2 ± 0.4
B5	262 ± 1	143 ± 23	66 ± 2	0.08 ± 0.02	2,056,000	7.2 ± 0.1	3.99 × 10^12^	50.7 ± 0.2	9.3 ± 0.5	3.9 ± 0.3

Characterization of PMMA/PDDA dispersions of high colloidal stability regarding particle size from DLS (Dz ± the mean standard deviation, SD) or from the SEM images (D ± the mean standard deviation), ζ values, P values, M_w_, SC, Np and elemental analysis for percentiles (%) of C, H and N

The effect of PDDA and MMA concentrations on the physical properties of the particles is in Table 1: Dz, ζ and the polydispersity (P) for the dispersions from dynamic light-scattering (DLS) were determined after exhaustive dialysis. In the case of formulations A3, A4, A5, B3, B4 and B5, the PDDA concentration is enough to give stability for the positively charged dispersions so that these are the formulations selected for additional characterization. Determinations of solid contents, conversion rate of monomer into polymer, particle number density (N_p_), average molecular weight (M_w_) obtained by size exclusion chromatography (SEC), morphology of lyophilized particles from scanning electron microscopy (SEM), and antimicrobial activity against bacteria from plating and colony forming unities (CFU) counting were carried out. Table 1 shows also data on N_p_ calculated from the mass of one particle (m_p_) taken as its volume (πr_p_^3^ 4/3) times the PMMA density (1.15 × 10^3^ g/dm^3^) where the particle radius (r_p_) is half the particle diameter (D) obtained by SEM. Since the solid content (SC) for each formulation is obtained by gravimetry in mg/mL and m_p_ is known, the particle number density N_p_ (the number of particles in 1 mL of dispersion) can be calculated (Table 1). The additional file 1 shows photographs (Additional file 1: Figure S1) and size distributions of NPs dispersions immediately after dialysis and synthesis (Additional file 1: Figure S2) or after two years (Additional file 1: Figure S3) plus data on molecular weights and PDI obtained from SEC (Additional file 1: Table S1).

The PDDA concentration in 1 mL of each dispersion was determined from the solid contents (SC), in mg/mL, and the % N determined by elemental analysis after lyophilization. One mole of PDDA contains 928.8 monomers since diallyldimethylammonium choride (DDA) molecular weight is 161.5 g and PDDA molecular weight is 150,000 g. Thus 928.8 mol of N in one mole of PDDA yields 13003.2 g N per mole of PDDA. The nitrogen mass in the lyophilized samples is the SC × F_N_ where F_N_ is the mass fraction of nitrogen atoms (N) in 1 mL of each dispersion and the PDDA concentration is given by:

$$[{\text{PDDA}}]({\text{mg/ml}}) = \frac{{1.5 \times 10^{5} \times {\text{SC}} \times {\text{F}}_{\text{N}} }}{13003.2}$$

The PDDA concentration for all dispersions is systematically smaller than the one added for the particle synthesis (Table 2). A possible reason for this might be related to the fact that PDDA is cationic and can adsorb onto the glass used for synthesis and also onto the cellulose acetate membrane where dialysis was performed. Therefore, The PDDA concentration used during the particle synthesis cannot be considered the real one present in the NPs. The incorporation of PDDA in the NPs is higher for the higher MMA concentrations used for particle synthesis (Table 2).

**Table 2:** Determining PDDA concentration in the NPs

Dispersion	% N	F_N_	SC (mg/mL)	[PDDA] (mg/mL)
A3	2.4	0.024	5.5	1.53
A4	1.5	0.015	10.1	1.74
A5	3.7	0.037	4.4	1.88
B3	3.0	0.030	7.3	2.52
B4	4.2	0.042	5.8	2.80
B5	3.9	0.039	7.2	3.24

Calculation of PDDA concentration in the hybrid PMMA/PDDA NPs from SC and % N. F_N_ is the mass fraction of nitrogen

**Fig. 1:**
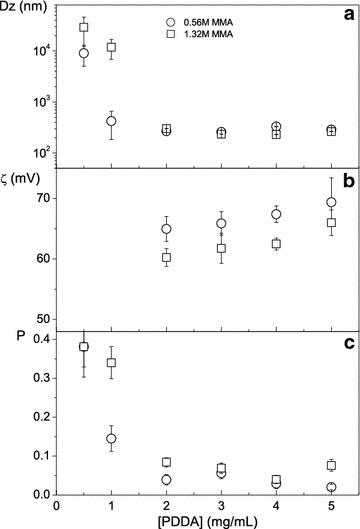
The dependence of physical properties of PMMA/PDDA NPs on PDDA concentration. **a** Dz as a function of PDDA concentration, **b** ζ as a function of PDDA concentration and, **c** P as a function of PDDA concentration, at two different MMA concentrations used for particle synthesis

The effect of PDDA concentration on Dz, ζ and P of the PMMA/PDDA NPs is in Fig. 1. At [PDDA] <2 mg/mL, the poor colloidal stability results in large sizes, precipitation, low ζ and high P. At [PDDA] >2 mg/mL, small sizes, high positive ζ and low P mean a good colloidal stability for the selected dispersions (A3, A4, A5, B3, B4, B5).

There is no aging effect on the physical properties of the selected dispersions. PMMA/PDDA formulations stored in the lab bench for 12 months at room temperature do not evidence precipitation/aggregation, flocculation, or apparent growth of microorganisms. The physical properties remain the same even 2 years after synthesis (Additional file 1: Figure S3).

**Fig. 2:**
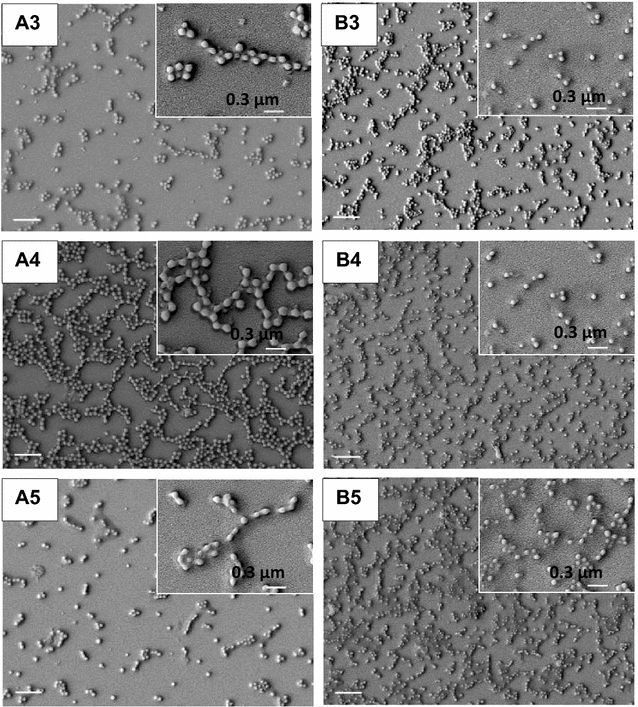
Morphology of PMMA/PDDA NPs from SEM micrographs. The dispersions A3, A4, A5, B3, B4 and B5 are seen under low and high magnification. The *bar* corresponds to 1 µm. The inserts show magnified images of the same dispersions

The NPs were also observed by SEM (Fig. 2). These images were submitted to the ImageJ software for determining D of the dried NPs (Table 1).

**Fig. 3:**
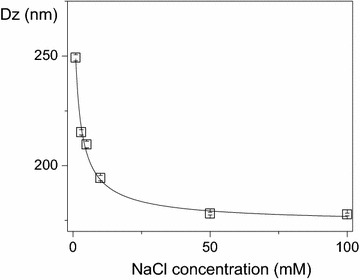
The collapse of the outer PDDA layer on NPs. Dz for NPs (A3 dispersion) is a function of NaCl concentration

The effect of ionic strength on Dz of NPs for the A3 dispersion is shown on Fig. 3. There is a reduction of Dz taking place as a function of the NaCl concentration in the NP medium. The Dz for A3 NPs decreases by ca. 70–80 nm due to the addition of 100 mM NaCl to the NP medium. On Table 1, the comparison between Dz and D suggests an interesting drying effect on the NPs structure. Dz for the A3 dispersion is 257 nm whereas D (obtained after drying) is 127 nm, meaning a reduction of ca. 130 nm after drying (Figs. 2, 3; Table 1). Drying or increasing the ionic strength of the medium substantially reduces the NPs diameter.

### Antimicrobial and hemolytic activity of PMMA/PDDA NPs

**Fig. 4:**
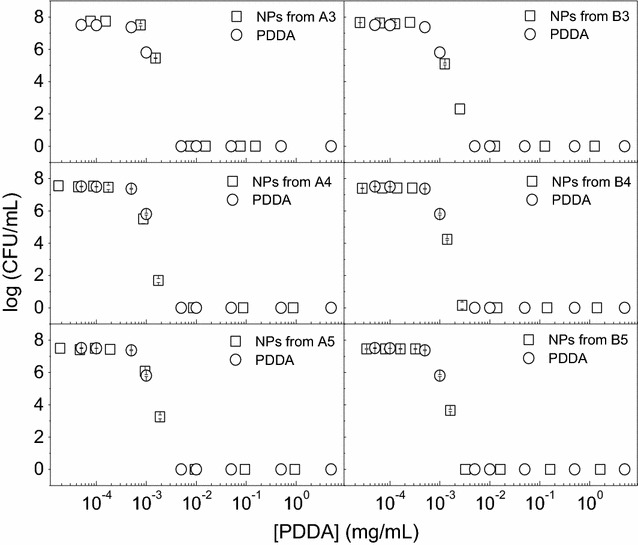
Antimicrobial activity of NPs against *E. coli*. Cell viability (log CFU/mL) for *E. coli* at 3–6 × 10^7^ CFU/mL as a function of PDDA concentration for free PDDA or PDDA in the PMMA/PDDA NPs diluted from dispersions A3, A4, A5, B3, B4 and B5. Cells and NPs or free PDDA interacted for 1 h before plating for CFU counting

**Fig. 5:**
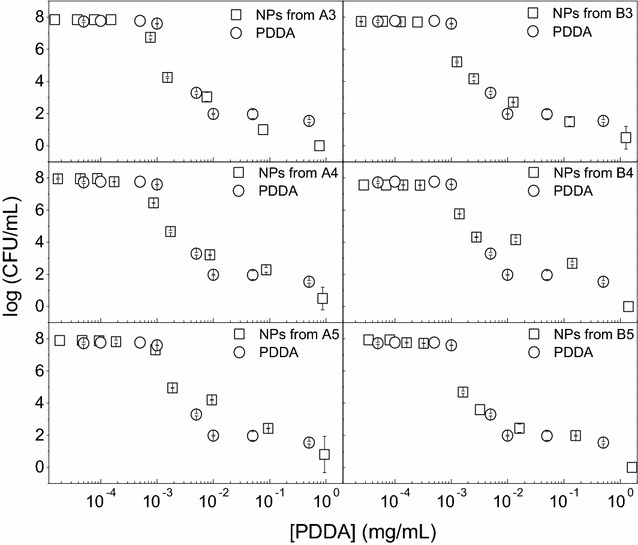
Antimicrobial activity of NPs against *S. aureus*. Cell viability (log CFU/mL) for *S. aureus* at 4–9 × 10^7^ CFU/mL as a function of PDDA concentration for free PDDA or PDDA in the PMMA/PDDA NPs diluted from dispersions A3, A4, A5, B3, B4 and B5. Cells and NPs or free PDDA interacted for 1 h before plating for CFU counting

**Fig. 6:**
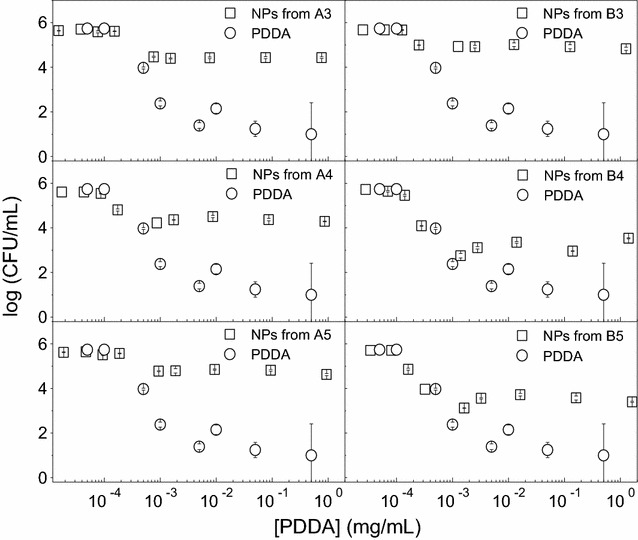
Antimicrobial activity of NPs against *C. albicans*. Cell viability (log CFU/mL) for *C. albicans* at 4–5 × 10^5^ CFU/mL as a function of PDDA concentration for free PDDA or PDDA in the PMMA/PDDA NPs diluted from dispersions A3, A4, A5, B3, B4 and B5. Cells and NPs or free PDDA interacted for 1 h before plating for CFU counting

The NPs of high colloidal stability were tested against the Gram–negative *E. coli* (Fig. 4), Gram-positive *S. aureus* (Fig. 5) and the yeast *C. albicans* (Fig. 6) revealing the effect of PDDA alone or in the PMMA/PDDA NPs on the cell viability of these microorganisms. One should firstly notice the logarithmic scale for the CFU/mL counting which allows the perfect determination of the potency and effectiveness of the antimicrobials under testing. The other way of presenting cell viability data, the percentile of CFU/mL, does not allow discriminating between moderate and highly potent antimicrobial agents. A two-log decrease of cell viability already shows on the percentile plot as an apparent decrease of cell viability to practically zero. PDDA and NPs are highly effective against *E. coli* reducing cell viability to practically zero (Fig. 4).

However, against *S. aureus*, PDDA concentrations leading to the complete loss of cell viability were much higher than those determined against *E. coli* (Fig. 5).

Against the yeast *C. albicans*, free PDDA is effective to kill almost completely the fungus but PDDA immobilized in the NPs has a reduced activity (Fig. 6), suggesting an important role for the PDDA mobility to reach the cell sites of action such as the cell membrane and the cell wall. On the PMMA/PDDA NPs, the PDDA link to the NP would hamper its mobility and thereby its activity against the fungus cells.

**Fig. 7:**
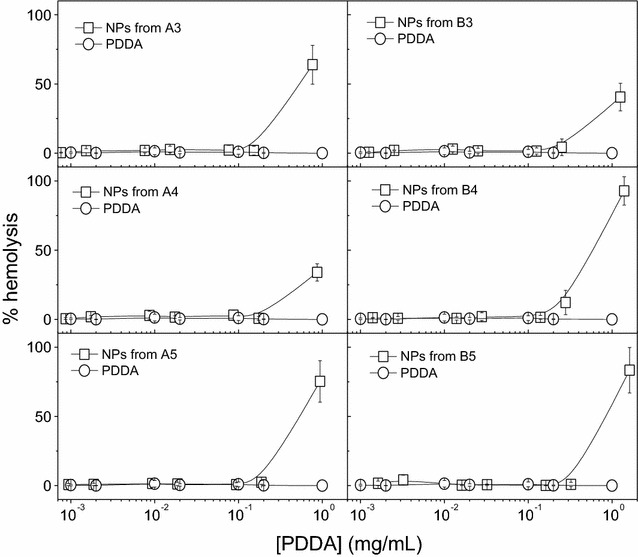
Toxicity of NPs against mammalian red blood cells. Hemolysis (%) as a function of PDDA concentration for free PDDA or PDDA in the PMMA/PDDA NPs diluted from dispersions A3, A4, A5, B3, B4 and B5. Red blood cells and NPs or PDDA interacted for 1 h before determining % hemolysis

PDDA alone barely affects the red blood cells over the range of PDDA concentrations for which PDDA kills efficiently the microorganisms (Fig. 7). However, with exception of the effect against *E. coli,* for PDDA in the NPs, the toxicity against the red blood cells is relevant over the same range of PDDA concentrations effective against the pathogenic microorganisms (Table 3).

**Table 3:** Antimicrobial and hemolytical activities of PMMA/PDDA NPs

Dispersion	MMC (% H)		
	*E. coli*	*S. aureus*	*C. albicans*
PDDA	0.005 (0)	>0.5 (0)	0.005 (0)^a^
A3	0.007 (0)	0.1 (15)	>0.8 (>63)
A4	0.007 (0)	0.5 (20)	>0.8 (>33)
A5	0.007 (0)	0.5 (48)	>1.0 (>75)
B3	0.010 (0)	0.6 (23)	>1.0 (>41)
B4	0.003 (0)	0.8 (65)	>1.5 (>90)
B5	0.003 (0)	0.8 (48)	>1.5 (>83)

MMC, in mg mL^−1^, and hemolysis percentiles in between parentheses (% H) at the MMC for free PDDA or in the PDDA/PMMA NPs

^a^For PDDA against *C. albicans*, MMC is the smallest [PDDA] leading to a five-log reduction of cell viability

## Discussion

### PMMA/PDDA NPs utility in vivo and ex vivo

The PMMA/PDDA NPs obtained by the one-pot synthesis are surfactant-free and have several interesting properties: NPs are all of the same size, have high colloidal stability and exhibit antimicrobial properties. These are remarkable properties achieved by a simple procedure as is emulsion polymerization. This method can possibly be carried out also in the presence of other interesting antimicrobial compounds aiming at their immobilization in the polymeric nanoparticles. The painting, coating, food processing, air-conditioning or water-treatment industries usually welcome antimicrobials for several ex vivo applications. The particle synthesis here described in the presence of PDDA can also be performed in the presence of other antimicrobial agents such as the Gemini surfactants [24–27], conjugated polyelectrolytes [28], polynorbornenes [29], lipopeptides [30], and hydrophobic drugs [31, 32], all of them representing good candidates for incorporation in PMMA nanoparticles during their synthesis. However, the characterization of the novel assemblies will have to be performed for each novel compound in the PMMA matrix and their activity compared to the one of the free antimicrobial substance.

### The collapse of the outer PDDA layer takes place upon drying or increasing the medium ionic strength

**Fig. 8:**
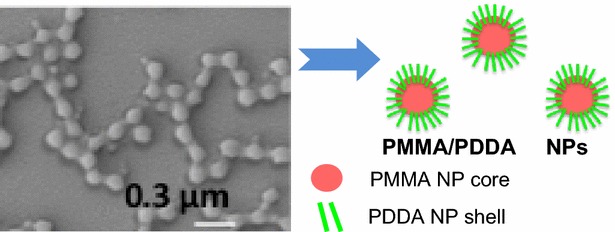
Schematic representation of the PMMA/PDDA NPs structure. The core–shell nature of the cationic PMMA/PDDA NPs at low ionic strength

The reduction in NP size taking place as a function of the NaCl concentration is meaningful (Fig. 3). This result suggests that neutralization of PDDA positive charges by monovalent salt collapses the stiff, protruding PDDA charged chains reducing the shell thickness on NPs. The effect of salt possibly is very similar to the effect of drying on the NP structure. The PDDA protuberances mechanically bound to the PMMA NP core have their charge screened by salt and their collapse amounts to a 80 nm reduction in particle size confirming the core–shell nature of the NPs structure, with PMMA residing in the core and PDDA occupying the shell. For any charged surface, the extent of the double layer thickness decreases with the electrolyte concentration, due to osmotic effects. For instance, for 10^−5^ M NaCl it is 100 nm, for 10^−3^ M NaCl it is 10 nm and for 10^−1^ M NaCl it is 1 nm. Additionally, the persistence length of a polyelectrolyte, such as PDDA, decreases as the ionic strength increases [33], so that at 1 mM NaCl the PDDA chains are stiff due to electrostatic repulsion among the segments, while at 100 mM NaCl the PDDA charges are screened and the chains behave like collapsed coils. Figure 8 illustrates the structure of the NPs suggested by the experiment shown in Fig. 3.

### The antimicrobial activity of PMMA/PDDA NPs depends on the microorganism and the hydrophobic–hydrophilic balance of the cationic polymer

The diverse antimicrobial effect of PMMA/PDDA NPs reducing eight-, seven- and two-logs of viable *E. coli*, *S. aureus* and *C. albicans* cells may be related to the differences in the microbial cell wall structures. A sensor system in *Staphylococcus* sp. is able to counteract the action of cationic antimicrobial compounds [34]. When these Gram-positive bacteria cells enter in contact with cationic compounds, the D-alanylation of teichoic acids and the lysylation of phosphatidylglycerol decrease the negative charge of the cell surface and membrane thereby hampering the adsorption of the cationic antimicrobials [34]. This might explain the relatively lower activity of the NPs against *S. aureus* (Fig. 5) when compared to the one against *E. coli* (Fig. 4). For the yeast cells, the antimicrobial compound has also to penetrate through the cell wall reaching the yeast cell membrane to exert fungicidal activity. The molecular architecture of the cell wall of *C. albicans* consists of an inner skeletal layer composed of the stress-bearing polysaccharides β-1,3-glucan and chitin, which run parallel to the cell surface [23]. The inner layer is kept together by extensive hydrogen bonding between individual β-1,3-glucan chains and by the β-1,3-glucan by a cross-linking protein. This three-dimensional skeletal network acts as a scaffold for a dense outer layer of glycoproteins extending into the environment forming like a brush layer. Our results suggest that the spherical cationic NPs would not be able to penetrate the brush layer yielding poor fungicidal activity for PMMA/PDDA NPs in comparison to the excellent activity of free PDDA, which can penetrate the brush layer unhindered due to its needle-like structure (Fig. 6). In fact, this result is consistent with previous data for PDDA as the outermost layer of self-assembled NPs in which PDDA motility to penetrate the cells is not restricted and the polymer can disassemble easily from the NP to penetrate the cell [35]. A good example that the mobility of the antimicrobial quaternary ammonium moiety indeed matters can be found in the dentistry field, where resins have been grafted with pendant and permanent quaternary ammonium groups [36]. Grafting of a quaternary ammonium antimicrobial component in the resin network can be achieved through copolymerization of the antimicrobial monomers with the conventional methacrylate monomers [36]. While free, non -polymerized quaternary ammonium monomers can rapidly kill oral pathogens, the antimicrobial component immobilized by poly-merization does not exhibit equally strong inhibitory effects [36].

For self-assembled NPs built from oppositely charged layers of cationic bilayer fragments, anionic polyelectrolyte and PDDA, the interaction of the outer PDDA layer with elements of the cell wall induce withdrawal of major components of the cell wall and membrane disruption culminating in leakage of intracellular components and microbe death [35]. Thus, freedom of the antimicrobial polymer to interact with the microbial cell is indeed very important.

The incorporation of antimicrobial surfactants or lipids in PMMA particles [13] or coatings [11, 12] during the PMMA synthesis by emulsion polymerization would represent a simple and inexpensive way of obtaining biocompatible antimicrobial materials. However, the construction of the PMMA/PDDA NPs with PDDA as the shell and PMMA as the core diminished the advantages of using PMMA as the biocompatible polymer (Figs. 3, 8). PDDA location in the NP shell leaves the antimicrobial toxic NP moiety as the outermost NP shell. Toxicity against the red blood cells becomes high as often described for cationic NPs and dendrimers [37]. The fact that free PDDA barely affects the viability of red blood cells (Fig. 7) is in agreement with previous work [23] and can be understood from simple adsorption of the PDDA macromolecule onto the red blood cell surface without penetration in the cell membrane. On the other hand, when compared with the free PDDA, the PMMA/PDDA NPs showed an increased ability to penetrate into the red blood cells disturbing the cell membrane and causing its lysis (Fig. 7). Previously described self-assembled NPs with PDDA as the outermost adsorbed layer are not hemolytic over the range of concentrations required to kill the microorganisms [23]. For the PMMA/PDDA NPs described in this work, this occurs only for *E. coli* (Fig. 4). For the other microorganisms, the relatively high microbicidal PDDA concentrations in the NPs belong to the same range of concentrations where hemolysis becomes significant (Table 3). When the cationic antimicrobial self-assembled NPs [21, 22] enter in contact with oppositely charged cells, we have recently shown that they disassemble and release PDDA to act as a free compound [35].

In vivo liposomes, particles and bacteria are engulfed by the phagocytic cells [38] so that their co-localization allows optimal activity for particle-based formulations against the microbes. The tests performed in vitro certainly cannot predict the efficacy of the in vivo PMMA/PDDA antimicrobial particles and further testing should be performed in animal models of infection.

**Fig. 9:**
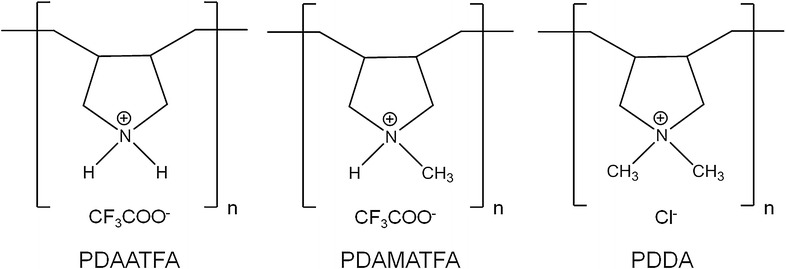
The chemical structure of three PDDA derivatives with increasing hydrophobic-hydrophilic balance. From *left* to *right* are PDAATFA, PDAMATFA and PDDA

Data on antimicrobial activity of some PDDA derivatives were previously reported by Timofeeva and coworkers [39]. These PDDA derivatives have none, one or two methyls on the nitrogen as shown in Fig. 9 for as poly (diallylammonium trifluoroacetate) (PDAATFA), poly (diallylmethylammonium trifluoroacetate) (PDAMATFA), and PDDA. On Table 4 the present results for antimicrobial activity are compared with those previously obtained by Timofeeva and coworkers [39]. Table 4 clarifies important aspects of the structure–activity relationship for PDDA and derivatives which varies in accordance to polymer molecular weight and hydrophobic-hydrophilic balance. The antimicrobial activity against Gram-negative bacteria increases with molecular weight and with the hydrophobic-hydrophilic balance of the cationic polymers. However, for Gram-positive bacteria or fungus these effects do not occur for all strains tested. In particular, against the fungus no effect of the molecular weight or the hydrophobic-hydrophilic balance are apparent. The fungus is very sensitive to all PDDA derivatives.

**Table 4:** The dependence of antimicrobial activity on the microorganism, M_w_ and hydrophobic–hydrophilic balance of the cationic polymer

Microorganism	MMC (µg/mL)					References
	PDAATFA		PDAMATFA, M_w_ (kDa)		PDDA	
	24	62	24	55	150	
*E. coli* ATCC 25922	125 ± 7.5^a^	15 ± 1.8^a^	62 ± 5.0^a^	7 ± 1.0^a^		[39]
					5	This work
*P. aeruginosa* ATCC 9027		125 ± 7.5^a^		31 ± 3.1^a^		[39]
*P. aeruginosa* MDR					1.5^b^	[35]
*K. pneumoniae* ATCC 13883		15 ± 1.8^a^		62 ± 5.0^a^		[39]
*K. pneumoniae* KPC+					0.9^b^	[35]
*S. aureus* ATCC 6538 P	31 ± 3.1^a^	1.5 ± 0.3^a^	62 ± 5.0^a^	7 ± 1.0^a^		[39]
*S. aureus* ATCC 29213					>500^b^	This work
MRSA					5.0^b^	[35]
*C. albicans* ATCC 865-653	3.5 ± 0.6^a^	1.5 ± 0.3^a^	3.5 ± 0.3^a^	3.5 ± 0.6^a^		[39]
*C. albicans* ATCC 90028					5	This work
*C. albicans* fluconazole resistant					0.8^b^	[35]

MMC, in mg mL^−1^, for PDAATFA, PDAMATFA and PDDA against bacteria and fungi. The M_w_ value is given in kDa. The hydrophobic–hydrophilic balance increases with methylation of the nitrogen. PDAATFA, PDAMATFA and PDDA have none, one or two methyl groups attached to the nitrogen, respectively

^a^The MMC is the minimal concentration for the total inhibition of microorganism growth

^b^The minimal concentration for reduction of CFU counting to one

## Conclusions

Several novel antimicrobial polymers are appearing in the literature, which require formulation to become biocompatible and less toxic against mammalian cells. A subtle balance between antimicrobial activity and toxicity against the mammalian cells must be achieved in novel formulations for antimicrobials. The novel formulations for antimicrobial polymers must ensure biocompatibility without hampering the antimicrobial activity. In this work, the physical immobilization of the antimicrobial polymer PDDA in biocompatible PMMA NPs preserves the PDDA activity against *E. coli* and its low toxicity against red blood cells, but the same does not occur against *S. aureus* and *C. albicans* due to their different cell walls that are less sensitive to cationic nanoparticles and very sensitive to the free antimicrobial polymer.

## Methods

### Chemicals

MMA, AIBN, NaCl and PDDA (20 weight % in water; 100,000–200,000 of molecular weight) were from Sigma-Aldrich (Steinheim, Germany) and were used without further purification. The syntheses were performed in a 1 mM NaCl solution prepared with Milli-Q water. The dispersions obtained by emulsion polymerization were purified by dialysis using cellulose acetate membranes (Sigma-Aldrich) with molecular weight cut-off in the range of 12,400 g/mol. Difco Mueller–Hinton Agar (MHA) was from Becton–Dickinson and Co (Sparks, MD, USA) and Sabouraud 4 % Glucose Agar (SA) was from Fluka Analytical (Sigma-Aldrich, Steinheim, Germany).

### Preparation of PMMA/PDDA nanoparticles by emulsion polymerization

Polymerization reactions were carried out at 85 °C under reflux for 2 h using 100 mL of aqueous solutions of NaCl 1 mM and PDDA over a range of PDDA concentrations at 0.56 or 1.32 M MMA as shown on Table 1. A weak flow of N_2_ was applied to the solution during 30 min both before and after adding 6 or 14 mL of MMA containing 0.036 g of AIBN initiator. After 2 h, the reaction mixture was removed from the dry bath and allowed to reach the room temperature (25 °C). The dispersions thus obtained were purified by dialysis against Milli-Q water until the conductivity of dialysis water achieved 5 µs/cm.

### Particle sizing and zeta-potential analysis of PMMA/PDDA dispersions

Size distributions, Dz, ζ and P were obtained by DLS using a Zeta Plus–Zeta Potential Analyzer (Brookhaven Instruments Corporation, Holtsville, NY, USA) equipped with a laser of 677 nm with measurements at 90°. P of the dispersions was determined by DLS following well defined mathematic equations [40]. Dz values were obtained from the log normal distribution of the light -scattered intensity curve against the diameter. ζ values were determined from the electrophoretic mobility (μ) and Smolukowski equation ζ = μη/ε, where η and ε are the viscosity and the dielectric constant of the medium, respectively. Samples were diluted 1:30 with a 1 mM NaCl water solution for performing the measurements at (25 ± 1) °C.

### Determination of PMMA molecular weight and particles morphology in PMMA/PDDA dispersions

The PMMA/PDDA dispersions were lyophilized in order to obtain dry particles. The conversion of monomer into polymer and the solid contents were determined by gravimetric measurements. The degree of polymerization was determined from the mean molecular weight divided by the monomer molecular weight (100, 12 g/mol). M_w_, pondered molecular weight (M_n_) and the polydispersity index (PDI) equal to M_w_/M_n_ were obtained by SEC using a Shimadzu HPLC/SEC class-VP equipped with two columns Viscogel™ (I-MBMMW 3078 and I-Oligo 3078, 30 cm × 8 mm each, Viscotek) with exclusion limits of 20 and 10 kDa, respectively, connected to a refractive index differential detector Shimadzu RID 10A. The solvent was chloroform with a flow of 1 mL/min. The SEC system was calibrated using seven polystyrene standards of low PDI (Aldrich/Waters; M_w_ = 820, 2460, 5120, 13,200, 29,300, 47,500 and 216,000 g/mol) and toluene was employed to identify the exclusion limit (conventional calibration). Samples of lyophilized PMMA/PDDA dispersions (10 ± 1 mg/mL) were firstly solubilized in chloroform with precipitation of the cationic PDDA and SEC analyses of the solubilized PMMA in the supernatant.

Elemental analyses were performed in a Perkin -Elmer CHN 200 equipment allowing the quantitative determination of carbon, hydrogen, and nitrogen in the lyophilized dispersions.

SEM was performed in a Jeol JSM-7401F equipment. 2μL of each dispersion was placed on silicon wafers and dried in a desiccator before being covered with a thin layer of gold for the SEM visualization. Mean D values from the SEM micrographs was also determined using the ImageJ software for particle size analysis and shown as a mean D ± mean standard deviation.

### Determination of the effect of ionic strength on Dz for NPs

The original A3 formulation obtained in pure water after dialysis was diluted 1:30 in aqueous NaCl solutions previously prepared over a range of NaCl concentrations (1–100 mM NaCl) and the mean hydrodynamic diameter was obtained as described above by dynamic light scattering. Dz values were plotted as a function of the final NaCl concentration (Additional file 1).

### Organisms and culture conditions

*E. coli* ATCC (American Type Culture Collection) 25322, *S. aureus* ATCC 29213 and *C. albicans* ATCC 90028 were reactivated from previously frozen stocks kept at −20 °C in appropriate storage medium. The bacterial strains were plated onto MHA before incubating the plates at 37 °C/18–24 h. The yeast cells were plated onto SA before incubating at 37 °C/48 h. Thereafter, some isolated colonies were transferred to an isotonic 0.264 M d-glucose solution, and the turbidity was adjusted to 0.5 of the McFarland scale [41]. The 0.264 M d-glucose solution was used instead of any culture medium because cationic molecules are inactivated by the relatively high ionic strength and also by the negatively charged molecules such as aminoacids and polysaccharides. For determination of cell viability in the presence of NPs, final bacteria and fungus cell concentrations in the suspensions were ca. 10^8^ and 10^5^ CFU/mL, respectively.

### Cell viability assays

Previous dilutions of the NPs over a range of NPs concentrations for obtaining the desired range of PDDA concentrations in the NPs were performed before interacting NPs and microbes. Microorganisms and dispersions (0.5 mL of each) interacted for 1 h at 25 °C before plating 0.1 mL of the diluted mixtures (dilution up to 100,000 times for bacteria and 1000 times for fungus) on agar in duplicate and incubating (24 h/37 °C for bacteria and 48 h/37 °C for fungus) for CFU counting. Cell viability was plotted as the log (CFU/mL) ± the mean standard deviation as a function of PDDA concentration in the control for PDDA only or PDDA in NP. The positive control was performed for mixtures of the microorganism suspension with the 0.264 M d-glucose solution. Minimal microbicidal concentration (MMC) is the lowest PDDA concentration required for complete killing of the microbes and log of cell viability equal to zero.

### Hemolysis assays

The defibrinated sheep blood for the hemolysis assay was centrifuged and the pelleted for rinsing three times in d-Glucose 0.264 M solution before being finally resuspended to yield a 1 % red blood cells suspension. An aliquot of 0.5 mL of the 1 % erythrocytes suspension interacted for 1 h with 0.5 mL of NPs dispersion before centrifuging (3 min/3000 rpm) and determining the supernatant absorbance at 514 nm (A). The negative control (A_C−_) was 0.5 mL 1 % erythrocytes suspension added of 0.5 mL 0.264 M d-glucose solution. The positive control (A_C+_) was 0.5 mL 1 % erythrocytes suspension added of 0.5 mL 1 % Triton-X in 0.264 M d-glucose solution. The percentile of hemolysis (%H) induced by PDDA alone or in NPs was obtained from %H = 100× (A − A_C−_)/A_C+_.

## Additional file

10.1186/s12951-015-0123-3 Figure S1. The photographs of the PMMA/PDDA dispersions synthesized by emulsion polymerization. Figure S2. The size distributions of NPs just after their synthesis and dialysis. Table S1. The size exclusion chromatography data for the stable NPs. Figure S3. The size distributions for the NPs 2 years after their synthesis.

## Abbreviations

PDDA: poly (diallyldimethylammonium) chloride
PMMA: poly (methylmethacrylate)
NPs: nanoparticles
MMA: methylmethacrylate
QACs: quaternary ammonium compounds
DODAB: dioctadecyldimethylammonium bromide
CTAB: cetyltrimethylammonium bromide
AIBN: azobisisobutyronitrile
Dz: zeta-average diameter
ζ: zeta-potential
P: polydispersity
DLS: dynamic light scattering
N_p_: particle number density
M_w_: average molecular weight
SEC: size exclusion chromatography
SEM: scanning electron microscopy
CFU: colony forming unities
m_p_: mass of one particle
r_p_: particle radius determined by SEM
D: particle diameter determined by SEM
SC: solid contents
DDA: diallyldimethylammonium chloride
F_N_: mass fraction of nitrogen atoms (N) in 1 mL of each dispersion
PDAATFA: poly (diallylammonium trifluoroacetate)
PDAMATFA: poly (diallylmethylammonium trifluoroacetate)
μ: electrophoretic mobility
η: viscosity of the medium
ε: dielectric constant of the medium
MHA: mueller-hinton agar
SA: sabouraud agar
M_n_: pondered molecular weight
PDI: polydispersity index equal to M_w_/M _n_
ATCC: American type culture collection
MMC: minimal microbicidal concentration
MDR: multidrug resistant microorganism
KPC+: strain producer of *K. pneumoniae* carbapenemase
MRSA: methicillin resistant *S. aureus*

## References

[1] Brooks BD, Brooks AE (2014). Therapeutic strategies to combat antibiotic resistance. Adv Drug Deliv Rev.

[2] Bertesteanu S, Chifiriuc MC, Grumezescu AM, Printza AG, Marie-Paule T, Grumezescu V, Mihaela V, Lazar V, Grigore R (2014). Biomedical applications of synthetic, biodegradable polymers for the development of anti-infective strategies. Curr Med Chem.

[3] Alvarez-Paino M, Muñoz-Bonilla A, López-Fabal F, Gómez-Garcés JL, Heuts JPA, Fernández-Garcia M (2015). Functional surfaces obtained from emulsion polymerization using antimicrobial glycosylated block copolymers as surfactants. Polym Chem..

[4] Carmona-Ribeiro AM, de Melo Carrasco LD (2013). Cationic antimicrobial polymers and their assemblies. Int J Mol Sci.

[5] Muñoz-Bonilla A, Fernández-Garcia M (2015). The roadmap of antimicrobial polymeric materials in macromolecular nanotechnology. Eur Polym J..

[6] Khaira GK, Ganguli A, Ghosh M (2014). Synthesis and evaluation of antibacterial activity of quaternized biopolymer from Klebsiella terrigena. J Appl Microbiol.

[7] Timofeeva L, Kleshcheva N (2011). Antimicrobial polymers: mechanism of action, factors of activity, and applications. Appl Microbiol Biotechnol.

[8] Ganewatta MS, Tang C (2015). Controlling macromolecular structures towards effective antimicrobial polymers. Polymer.

[9] Taresco V, Crisante F, Francolini I, Martinelli A, D’Ilario L, Ricci-Vitiani L, Buccarelli M, Pietrelli L, Piozzi A (2015). Antimicrobial and antioxidant amphiphilic random copolymers to address medical device-centered infections. Acta Biomater.

[10] Xue Y, Xiao H, Zhang Y (2015). Antimicrobial polymeric materials with quaternary ammonium and phosphonium salts. Int J Mol Sci.

[11] Pereira EM, Kosaka PM, Rosa H, Vieira DB, Kawano Y, Petri DF, Carmona-Ribeiro AM (2008). Hybrid materials from intermolecular associations between cationic lipid and polymers. J Phys Chem B..

[12] Melo LD, Palombo RR, Petri DF, Bruns M, Pereira EM, Carmona-Ribeiro AM (2011). Structure-activity relationship for quaternary ammonium compounds hybridized with poly(methyl methacrylate). ACS Appl Mater Interfaces..

[13] Naves AF, Palombo RR, Carrasco LD, Carmona-Ribeiro AM (2013). Antimicrobial particles from emulsion polymerization of methyl methacrylate in the presence of quaternary ammonium surfactants. Langmuir.

[14] Gan LM, Chew CH, Ng SC, Loh SE (1993). Polymerization of methyl methacrylate in ternary systems: emulsion and microemulsion. Langmuir.

[15] El-Asser MS. Scientific methods for the study of polymer colloids and their applications. In: Candau F, Ottewill RH, editors. London: Kluwer, Academic Publishers. 1990.

[16] Harkins WD (1947). A general theory of the mechanism of emulsion polymerization. J Am Chem Soc.

[17] Lichti G, Gilbert RG, Napper DH (1983). Mechanisms of latex particle formation and growth in the emulsion polymerization of styrene using the surfactant sodium dodecyl sulfate. J Polym Sci..

[18] Feeney PJ, Napper DH, Gilbert RG (1984). Coagulative nucleation and particle size distributions in emulsion polymerization. Macromolecules.

[19] Capek I (2001). Microemulsion polymerization of styrene in the presence of a cationic emulsifier. Adv Colloid Interface Sci.

[20] Antonietti M, Lohmann S, Van Niel C (1992). Polymerization in microemulsion. 2. Surface control and functionalization of microparticles. Macromolecules.

[21] Vieira DB, Carmona-Ribeiro AM (2008). Cationic nanoparticles for delivery of amphotericin B: preparation, characterization and activity in vitro. J Nanobiotechnol.

[22] Melo LD, Mamizuka EM, Carmona-Ribeiro AM (2010). Antimicrobial particles from cationic lipid and polyelectrolytes. Langmuir.

[23] Carmona-Ribeiro AM, Carrasco LDM (2013). Fungicidal assemblies and their mode of action. OA Biotechnol..

[24] Shirai A, Ueta S, Maseda H, Kourai H, Omasa T (2012). Action of reactive oxygen species in the antifungal mechanism of gemini-pyridinium salts against yeast. Biocontrol Sci..

[25] Yarinich LA, Burakova EA, Zakharov BA, Boldyreva EV, Babkina IN, Tikunova NV, Silnikov VN (2015). Synthesis and structure-activity relationship of novel 1,4-diazabicyclo[2.2.2]octane derivatives as potent antimicrobial agents. Eur J Med Chem.

[26] Hoque J, Akkapeddi P, Yarlagadda V, Uppu DS, Kumar P, Haldar J (2012). Cleavable cationic antibacterial amphiphiles: synthesis, mechanism of action, and cytotoxicities. Langmuir.

[27] Tavano L, Infante MR, Riya MA, Pinazo A, Vinardell MP, Mitjans M, Manresa MA, Perez L (2013). Role of aggregate size in the hemolytic and antimicrobial activity of colloidal solutions based on single and Gemini surfactants from arginine. Soft Matter.

[28] Wang Y, Corbitt TS, Jett SD, Tang Y, Schanze KS, Chi EY, Whitten DG (2012). Direct visualization of bactericidal action of cationic conjugated polyelectrolytes and oligomers. Langmuir.

[29] Ilker MF, Nüsslein K, Tew GN, Coughlin EB (2004). Tuning the hemolytic and antibacterial activities of amphiphilic polynorbornene derivatives. J Am Chem Soc.

[30] Findlay B, Zhanel GG, Schweizer F (2012). Investigating the antimicrobial peptide ‘window of activity’ using cationic lipopeptides with hydrocarbon and fluorinated tails. Int J Antimicrob Agents.

[31] Jain K, Verma AK, Mishra PR, Jain NK (2015). Characterization and evaluation of amphotericin B loaded MDP conjugated poly (propylene imine) dendrimers. Nanomedicine..

[32] Mohamed-Ahmed AH, Les KA, Seifert K, Croft SL, Brocchini S (2013). Noncovalent complexation of amphotericin-B with Poly(α-glutamic acid). Mol Pharm.

[33] Barrat J, Joanny JF (1993). Persistence length of polyelectrolyte chains. Europhys Lett.

[34] Otto M (2009). *Staphylococcus epidermidis*—the “accidental” pathogen. Nat Rev Microbiol.

[35] Carrasco LD, Sampaio JL, Carmona-Ribeiro AM (2015). Supramolecular cationic assemblies against multidrug-resistant microorganisms: activity and mechanism of action. Int J Mol Sci.

[36] Imazato S, Chen J, Ma S, Izutani N, Li F (2012). Antibacterial resin monomers based on quaternary ammonium and their benefits in restorative dentistry. Jpn Dent Sci Rev..

[37] Thiagarajan G, Greish K, Ghandehari H (2013). Charge affects the oral toxicity of poly(amidoamine) dendrimers. Eur J Pharm Biopharm.

[38] Bakker-Woudenberg IA, Schiffelers RM, Storm G, Becker MJ, Guo L (2005). Long-circulating sterically stabilized liposomes in the treatment of infections. Methods Enzymol.

[39] Timofeeva LM, Kleshcheva NA, Moroz AF, Didenko LV (2009). Secondary and tertiary polydiallylammonium salts: novel polymers with high antimicrobial activity. Biomacromolecules.

[40] Grabowski E, Morrison I. Measurements of suspended particles by quasi-elastic light scattering. In: Dahneke B, editor. New York: Wiley. 1983. pp 199–236.

[41] Chapin KC, Lauderdale T, Murray PR, Baron EJ, Jorgensen JH, Landry ML, Pfaller MA (2007). Reagents, stains, and media: bacteriology. Manual of clinical microbiology.

