# Supplementary material for: The antimicrobial activity of free and immobilized poly (diallyldimethylammonium) chloride in nanoparticles of poly (methylmethacrylate)
Source: J Nanobiotechnology. 2015 Sep 24;13:58. doi: 10.1186/s12951-015-0123-3 (PMC4582890; doi:10.1186/s12951-015-0123-3)
Supplement: Supplementary file 1 — 10.1186/s12951-015-0123-3 Figure S1. The photographs of the PMMA/PDDA dispersions synthesized by emulsion polymerization. Figure S2. The size distributions of NPs just after their synthesis and dialysis. Table S1. The size exclusion chromatography data for the stable NPs. Figure S3. The size distributions for the NPs 2 years after their synthesis. [file 12951_2015_123_MOESM1_ESM.pdf]

# The antimicrobial activity of free and immobilized poly (diallyldimethylammonium) chloride in nanoparticles of poly (methylmethacrylate)

*Luccas Missfeldt Sanches,*[1] *Denise Freitas Siqueira Petri,*[2] *Letícia Dias de Melo Carrasco*[1,3] *and Ana Maria Carmona-Ribeiro*[1,3]*

***Corresponding Author:** Ana Maria Carmona-Ribeiro. Biocolloids Lab, Departamento de Bioquímica, Instituto de Química, Universidade de São Paulo, Caixa Postal 26077, CEP 05513-970, São Paulo SP, Brazil.

[1]Biocolloids Lab, Departamento de Bioquímica, Instituto de Química, Universidade de São Paulo, Caixa Postal 26077, CEP 05513-970, São Paulo SP, Brazil.

[2]Instituto de Química, Universidade de São Paulo, Caixa Postal 26077, CEP 05513-970, São Paulo SP, Brazil.

[3]Departamento de Análises Clínicas e Toxicológicas, Faculdade de Ciências Farmacêuticas, Universidade de São Paulo, CEP 05508-900 São Paulo, Brazil.

**Additional file**

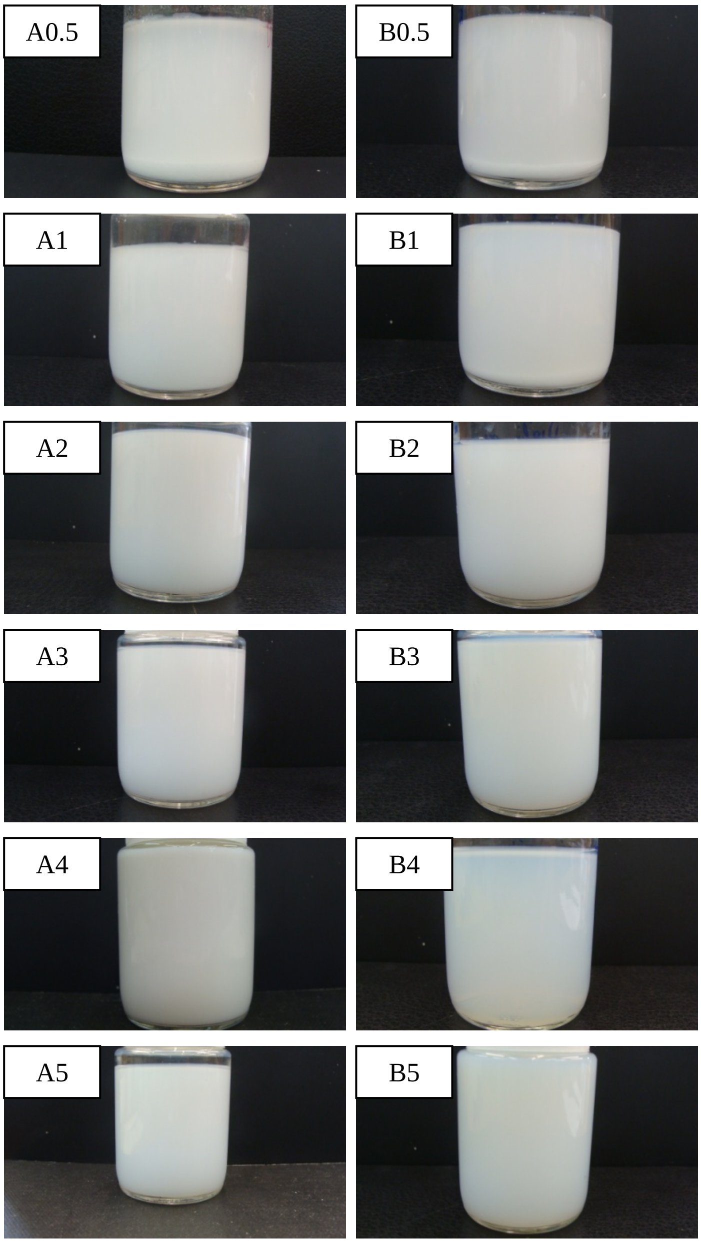


**Fig. S1** Photographs of NPs dispersions immediately after dialysis and synthesis.

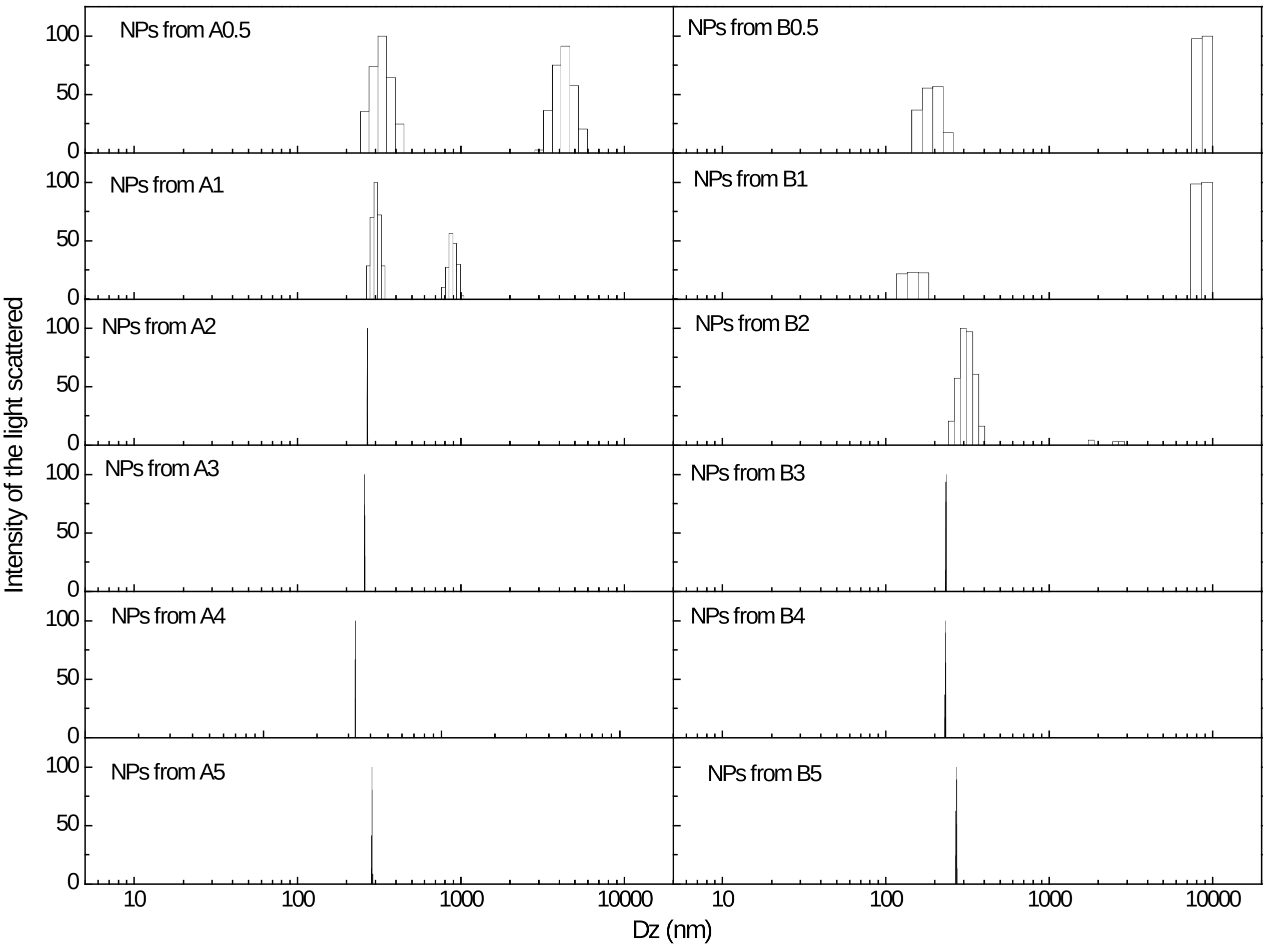


**Fig. S2** Intensity of the light scattered as a function of Dz (nm) for NPs immediately after synthesis and dialysis.

**Table S1** Data from size exclusion chromatography for determining $M_w$, $M_n$, PDI and the degree of polymerization (DP). As products of the polymerization, chains with different lengths are formed so that there will be a distribution of molecular weights. Two molecular weights can be determined from the distributions: the average molecular weight ($M_w$), that is, the total weight of polymer divided by the total number of polymer molecules (n), and the pondered molecular weight ($M_n$), which corresponds to the pondered sum of molecular weights divided by the total weight.

| NPs | $M_w$ (g/mol) | $M_n$ (g/mol) | PDI | DP |
|---|---|---|---|---|
| **A3** | 1,173,000 | 810643 | 1.447 | 11716 |
| **A4** | 1,065,000 | 430129 | 2.476 | 10650 |
| **A5** | 2,872,000 | 1752288 | 1.639 | 28686 |
| **B3** | 1,514,000 | 933416 | 1.622 | 15122 |
| **B4** | 2,362,000 | 1711594 | 1.380 | 23592 |
| **B5** | 2,056,000 | 1113759 | 1.846 | 20535 |

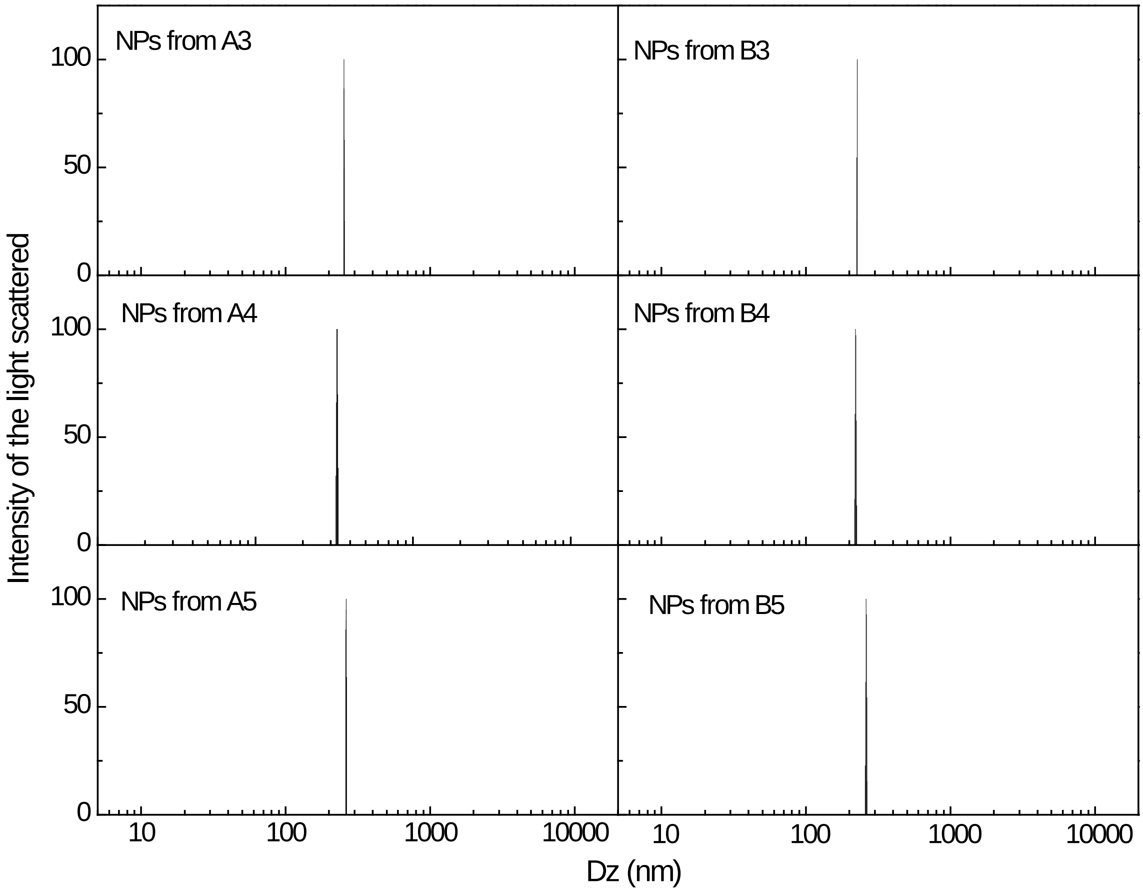


**Fig. S3** Intensity of the light scattered as a function of Dz (nm) for NPs 2 years after the synthesis. The data illustrate the high colloidal stability of the dispersions.
